# Independent and joint associations of social participation and depressive symptoms with incident stroke risk in older adults: a prospective cohort study based on CHARLS and ELSA

**DOI:** 10.1186/s12889-026-28217-z

**Published:** 2026-06-26

**Authors:** Liangze Ma, Yukun Ma, Zixun Huang, Lincui Wang, Kaixuan Ma, Ze Lin, Jianhong Li, Lingbin He, Chunhong Ren, Xin Zhang

**Affiliations:** 1 Shantou University Medical College, Shantou, 515041 China; 2 Laboratory of Molecular Cadology, Department of Cardiology, The First Affiliated Hospital of Shantou University Medical College, Shantou, 515041 China; 3 The First Affiliated Hospital of Shantou University Medical College, Shantou, 515041 China; 4 International Medical Service Center, The First Affiliated Hospital of Shantou University Medical College, Shantou, 515041 China; 5 Cancer Hospital of Shantou University Medical College, Shantou, 515041 China

**Keywords:** Social participation, Depressive symptoms, Incident stroke, Older adults, CHARLS, ELSA

## Abstract

**Background:**

Stroke remains a leading cause of death and disability among older adults. Depressive symptoms and low social participation have been linked to stroke risk via behavioral and biological pathways. This study examined the independent and joint associations of social participation (SP) and depressive symptoms (DS) with incident stroke in two nationally representative cohorts.

**Methods:**

Data were derived from two nationally representative cohorts: the China Health and Retirement Longitudinal Study (CHARLS; 2011–2018) and the English Longitudinal Study of Ageing (ELSA; 2012–2019). SP and DS were assessed at baseline and categorized into four phenotypic subgroups (SP+/DS−, SP−/DS−, SP+/DS+, SP−/DS+). Cox proportional hazards models were fitted within each cohort with sequential covariate adjustment. To address missing data, sensitivity analyses were performed using the multiple imputation method (MICE).

**Results:**

In CHARLS (*n* = 10,555; mean age 58.3 years), DS + was associated with elevated stroke risk (Model 3 h = 1.40, 95% CI 1.18–1.65, *p* < 0.001), whereas SP− showed no significant association (HR = 1.14, 95% CI 0.96–1.34, *p* = 0.13). Compared with the SP+/DS− reference phenotype, the SP−/DS+ joint phenotype was associated with an increased stroke risk (HR = 1.56, 95% CI 1.27–1.93, *p* < 0.001). In ELSA (*n* = 5,321; mean age 65.1 years), SP − was not associated with stroke risk (HR = 1.05, 95% CI 0.74–1.51, *p* = 0.78), and DS+ exhibited a similar but non-significant association (HR = 1.54, 95% CI 0.95–2.51, *p* = 0.08); the SP−/DS + was associated with elevated stroke risk (HR = 1.87, 95% CI 1.04–3.37, *p* = 0.04).

**Conclusions:**

Depressive symptoms were associated with incident stroke, whereas low social participation alone showed no consistent association with incident stroke. Notably, the joint phenotype SP−/DS+ identified a subgroup with the highest incident stroke risk, although such associations exhibited some heterogeneity between cohorts.

## Introduction

Stroke remains a leading cause of death and long-term disability worldwide, imposing a substantial burden with marked regional heterogeneity [1–3]. Given the severe long-term functional impairment and ongoing care needs after stroke, enhancing primary prevention and identifying modifiable risk factors represent key priorities for public health and clinical practice [4–6]. Beyond established cardiovascular risk factors (e.g., hypertension, diabetes, and smoking), psychosocial factors are increasingly recognized as critical components of stroke risk stratification and comprehensive prevention frameworks [4, 7].

Depressive symptoms (DS) are prevalent among middle-aged and older adults and contribute substantially to functional impairment and population health burden [8, 9]. Meta-analyses of community-dwelling older adults have reported a considerable burden of late-life depressive symptoms, with prevalence estimates of approximately 19.5% for dimensional depression and 16.5% for lifetime major depressive disorder [8–10, 11].

Prospective evidence consistently links depression with elevated stroke risk. Systematic reviews and meta-analyses have shown that depression is associated with higher risks of incident stroke and stroke-related mortality [12, 13], and regional cohort studies have reported similar associations between depressive symptoms and stroke [14–17]. Moreover, studies incorporating repeated symptom assessments suggest that persistently elevated depressive symptom trajectories may further identify individuals at increased stroke risk [18, 19]. Potential pathways linking depression to stroke risk include inflammatory, neuroendocrine, vascular, and behavioral processes [20–23].

Beyond depression, social participation (SP) and broader social connectedness are increasingly recognized as pertinent to cardiovascular health [24–26]. Large prospective cohort studies have linked social isolation and loneliness with heightened risks of cardiovascular events, including stroke. However, estimates often attenuate after adjustment for conventional risk factors, suggesting that health behaviours and healthcare access may partly shape these associations [27, 28]. Longitudinal evidence also suggests that persistent loneliness may be relevant to incident stroke risk [28, 29].

Despite the accumulating evidence, important knowledge gaps persist. First, depressive symptoms and adverse social exposures frequently co-occur in later life [30, 31], yet most existing stroke literature assesses these two factors in isolation. It remains unclear whether their combined phenotype identifies stroke risk patterns beyond either single factor alone [12, 32].

Second, most available evidence on social determinants of stroke focuses on relationship-deficit constructs, such as loneliness and social isolation [32, 33]. In contrast, less attention has been paid to behavior-oriented insufficient social participation, which may be more directly modifiable [31].

Third, cross-national comparisons using harmonised definitions and comparable analytic strategies remain scarce, although social structure, welfare systems, and cultural norms may shape social participation patterns and their links with depression-related stroke risk [34].

Therefore, using data from the China Health and Retirement Longitudinal Study (CHARLS) [35] and the English Longitudinal Study of Ageing (ELSA) [36], we examined the independent and joint associations of insufficient social participation and depressive symptoms with incident stroke. By applying harmonised exposure definitions and parallel analytic approaches across cohorts, we assessed whether the co-occurrence identifies a higher-risk psychosocial phenotype across distinct sociocultural settings [5, 24].

## Methods

**Fig. 1:**
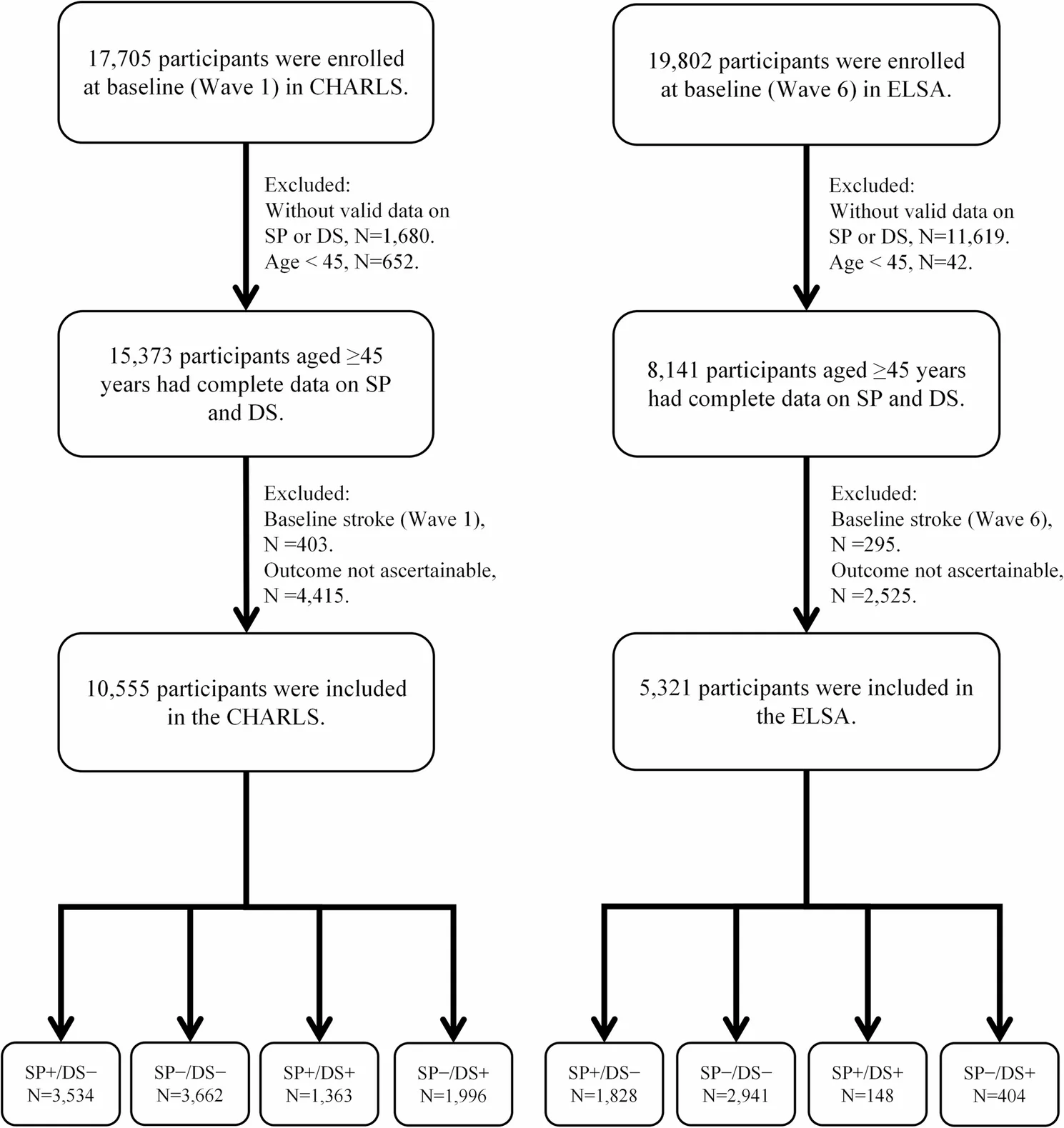
The overall flowchart of the study. Study flowchart and sample selection in CHARLS and ELSA. SP+ indicates participation in ≥ 1 social activity at baseline, SP− indicates no participation in any activity, DS+ indicates CES-D score above the cut-point (CHARLS: CES-D-10 ≥ 10; ELSA: CES-D-8 ≥ 4), DS− indicates below the cut-point

In this prospective cohort study, parallel analyses were conducted using two nationally representative ageing cohorts: CHARLS [35] and ELSA [36]. For CHARLS, the 2011–2012 national baseline survey was defined as the baseline time point, with follow-up interviews carried out in 2013, 2015, and 2018. For ELSA, Wave 6 (2012–2013) was defined as baseline, with follow-up conducted through Waves 7 (2014–2015), 8 (2016–2017), and 9 (2018–2019). In both cohorts, participants were eligible for inclusion if they had no history of stroke at baseline. Participants with missing data on key exposures or the outcome were excluded from the primary analyses. Follow-up continued until the first reported stroke event, loss to follow-up, death, or the last available follow-up visit, whichever occurred first. The sample selection process is detailed in Fig. 1.

### Exposure assessment

Two primary exposures were evaluated: social participation (SP) and depressive symptoms (DS). A four-category joint exposure phenotype was constructed for analysis. Social participation (SP) was harmonized across the two cohorts using questionnaire items covering social, leisure, and group activities: participants engaging in ≥ 1 activity at baseline were classified as SP+, whereas those with no participation were classified as SP−. Depressive symptoms (DS) were assessed using the Center for Epidemiologic Studies Depression Scale (CES-D), with cohort-specific cut-offs applied. Specifically, CHARLS used the 10-item CES-D (DS+ defined as a total score ≥ 10), whereas ELSA used the 8-item CES-D (DS+ defined as a total score ≥ 4). Participants were classified into four mutually exclusive groups: SP+/DS− (reference), SP−/DS−, SP+/DS+, and SP−/DS+.

Outcome Ascertainment and Event Timing

The primary outcome was incident stroke during follow-up. At each follow-up wave, stroke status was ascertained based on self-reported physician diagnosis. Incident stroke was defined as the first reported stroke at any follow-up wave among participants without stroke at baseline. Because exact dates of stroke onset were not available in the harmonized datasets used for this analysis, event time was approximated using the calendar year assigned to the wave in which stroke was first reported (CHARLS: 2011, 2013, 2015, and 2018; ELSA: 2012, 2014, 2016, and 2019). Follow-up time was calculated as the difference between the baseline year and the assigned event year; participants without stroke were censored at the last follow-up year (CHARLS: 2018; ELSA: 2019).

### Covariates

Covariates were harmonized across cohorts and included age, sex, marital status, education level, smoking status, alcohol drinking status, household economic resources (e.g., household expenditure/consumption or income), body mass index (BMI), baseline chronic disease burden summarized as the number of self-reported physician-diagnosed chronic conditions (0, 1, or ≥ 2).

### Statistical analysis

Cox proportional hazards regression models were fitted separately in CHARLS and ELSA to estimate hazard ratios (HRs) and 95% confidence intervals (95% CIs) for incident stroke. Associations were examined for social participation (SP) and depressive symptoms (DS) individually, as well as for the four-category joint exposure phenotype (SP+/DS − as the reference group; SP−/DS−, SP+/DS+, and SP−/DS+). Covariates were entered sequentially to address confounding. Model 1 was unadjusted. Model 2 was adjusted for key demographic and socioeconomic factors, including age, sex, education, marital status, and household economic resources. Model 3 was further adjusted for health behaviors and baseline health status, including smoking, alcohol consumption, body mass index, and chronic disease burden, for participants with complete data on these measures. These sequential models were used to test the robustness of the associations under increasing adjustment. Model 3 is interpreted as a fully adjusted conditional association model. Pre-specified subgroup analyses were performed for the joint-exposure phenotype by fitting stratified Cox models and estimating multiplicative interaction using exposure-by-subgroup terms. Missing data were handled using multiple imputation by chained equations (MICE) in pre-specified sensitivity analysis, and Cox models were refitted in the imputed datasets.

All tests were two-sided, with statistical significance set at *p* < 0.05. All analyses were performed using R version 4.4.1.

## Results

### Participant characteristics

CHARLS included 10,555 middle-aged and older adults without stroke at baseline (mean age, 58.3 ± 8.8 years). Across the four SP/DS categories, the distribution was 33.5% (SP+/DS−), 34.7% (SP−/DS−), 12.9% (SP+/DS+), and 18.9% (SP−/DS+). Stroke incidence increased across categories: 8.0% in the reference group (SP+/DS−), 8.2% in SP−/DS−, 10.6% in SP+/DS+, and 11.8% in SP−/DS+ (Table 1). Compared with the reference group, participants in the SP−/DS+ group were older, had lower educational attainment, and had a higher baseline chronic disease burden (Table 1). ELSA included 5,321 older adults without stroke at baseline (mean age, 65.1 ± 8.4 years). Across the four SP/DS categories, the distribution was 34.4% (SP+/DS−), 55.3% (SP−/DS−), 2.8% (SP+/DS+), and 7.6% (SP−/DS+) (Table 1). Stroke incidence was 3.7% in the reference group (SP+/DS−) and 5.7% in the SP−/DS+ group. In ELSA, the SP+/DS+ subgroup comprised only 148 participants with 6 incident stroke cases, resulting in limited statistical precision; therefore, non-significant effect estimates for this subgroup should be interpreted cautiously.

**Table 1:** Baseline characteristics of participants in CHARLS and ELSA

Characteristics	CHARLS (*n* = 10555)					ELSA (*n* = 5321)				
	SP+/DS−	SP−/DS−	SP+/DS+	SP−/DS+	Overall	SP+/DS−	SP−/DS−	SP+/DS+	SP−/DS+	Overall
Total	3534	3662	1363	1996	10,555	1828	2941	148	404	5321
Age, mean (S.D.)	57.5 (9.0)	58.2 (8.7)	59.0 (9.0)	59.3 (8.6)	58.3 (8.8)	65.6 (8.2)	65.1 (8.5)	64.8 (9.3)	64.0 (8.7)	65.1 (8.4)
Sex, n (%)										
Female	1726 (48.8)	1774 (48.4)	902 (66.2)	1273 (63.8)	5675 (53.8)	929 (50.8)	1652 (56.2)	109 (73.6)	277 (68.6)	2967 (55.8)
Male	1808 (51.2)	1888 (51.6)	461 (33.8)	723 (36.2)	4880 (46.2)	899 (49.2)	1289 (43.8)	39 (26.4)	127 (31.4)	2354 (44.2)
Marital status, n (%)										
Married and living with spouse	3064 (86.7)	3221 (88.0)	1041 (76.4)	1646 (82.5)	8972 (85.0)	1364 (77.5)	2106 (75.3)	91 (62.8)	220 (56.6)	3781 (74.3)
Married but living without spouse	160 (4.5)	110 (3.0)	93 (6.8)	66 (3.3)	429 (4.1)	—	—	—	—	—
Single/divorced/widowed	310 (8.8)	331 (9.0)	229 (16.8)	284 (14.2)	1154 (10.9)	396 (22.5)	690 (24.7)	54 (37.2)	169 (43.4)	1309 (25.7)
Education level, n (%)										
Elementary school or below	2080 (58.9)	2498 (68.2)	1030 (75.6)	1602 (80.3)	7210 (68.3)	239 (13.9)	831 (30.8)	36 (26.1)	151 (41.1)	1257 (25.6)
Middle school or above	1454 (41.1)	1164 (31.8)	333 (24.4)	394 (19.7)	3345 (31.7)	1476 (86.1)	1863 (69.2)	102 (73.9)	216 (58.9)	3657 (74.4)
Residence, n (%)										
Rural	2051 (58.0)	2366 (64.6)	997 (73.1)	1440 (72.1)	6854 (64.9)	—	—	—	—	—
Urban	1483 (42.0)	1296 (35.4)	366 (26.9)	556 (27.9)	3701 (35.1)	1828 (100.0)	2941 (100.0)	148 (100.0)	404 (100.0)	5321 (100.0)
Smoking status, n (%)										
Non-smoker	2037 (57.6)	2202 (60.1)	907 (66.5)	1333 (66.8)	6479 (61.4)	834 (45.6)	1119 (38.0)	65 (43.9)	143 (35.4)	2161 (40.6)
Smoker	1497 (42.4)	1460 (39.9)	456 (33.5)	663 (33.2)	4076 (38.6)	994 (54.4)	1822 (62.0)	83 (56.1)	261 (64.6)	3160 (59.4)
Drinking status, n (%)										
Drinker	1141 (34.1)	1040 (30.0)	322 (24.7)	432 (22.6)	2935 (29.3)	1674 (92.4)	2595 (89.4)	123 (83.1)	313 (78.4)	4705 (89.4)
Non-drinker	2202 (65.9)	2427 (70.0)	980 (75.3)	1479 (77.4)	7088 (70.7)	137 (7.6)	308 (10.6)	25 (16.9)	86 (21.6)	556 (10.6)
Number of chronic conditions, n (%)										
≥ 2	761 (21.5)	791 (21.6)	567 (41.6)	795 (39.8)	2914 (27.6)	739 (40.4)	1178 (40.1)	84 (56.8)	260 (64.4)	2261 (42.5)
1	1085 (30.7)	1139 (31.1)	429 (31.5)	654 (32.8)	3307 (31.3)	589 (32.2)	991 (33.7)	36 (24.3)	88 (21.8)	1704 (32.0)
0	1688 (47.8)	1732 (47.3)	367 (26.9)	547 (27.4)	4334 (41.1)	500 (27.4)	772 (26.2)	28 (18.9)	56 (13.9)	1356 (25.5)
Incident stroke, n (%)										
No	3250 (92.0)	3363 (91.8)	1218 (89.4)	1760 (88.2)	9591 (90.9)	1761 (96.3)	2836 (96.4)	142 (95.9)	381 (94.3)	5120 (96.2)
Yes	284 (8.0)	299 (8.2)	145 (10.6)	236 (11.8)	964 (9.1)	67 (3.7)	105 (3.6)	6 (4.1)	23 (5.7)	201 (3.8)
Annual household expenditure, mean (S.D.)	8102.4 (11259.4)	6357.5 (7138.9)	6710.8 (8838.8)	6059.3 (8153.6)	6940.0 (9158.4)	8942.4 (42689.7)	5637.4 (30947.2)	8305.4 (45475.1)	4261.6 (40785.6)	6741.1 (36602.0)
BMI, mean (S.D.)	24.1 (3.8)	23.4 (3.8)	23.4 (4.0)	23.0 (3.9)	23.6 (3.9)	28.1 (4.8)	28.2 (5.1)	28.5 (6.1)	29.5 (6.3)	28.3 (5.1)
Follow-up time, mean (S.D.)	7.0 (0.5)	6.9 (0.6)	6.9 (0.5)	6.9 (0.7)	6.9 (0.6)	5.9 (0.5)	5.9 (0.5)	5.9 (0.6)	5.9 (0.6)	5.9 (0.5)

*S.D* standard deviation, *CHARLS* China Health and Retirement Longitudinal Study, *ELSA* English Longitudinal Study of Ageing, *BMI* Body Mass Index, *SP+* participation in ≥ 1 social activity at baseline, *SP −* no participation in any activity, *DS + CES-D* score above the cut-point (CHARLS: CES-D-10 ≥ 10; ELSA: CES-D-8 ≥ 4), *DS−* below the cut-point

### Independent associations of SP and DS with incident stroke

**Fig. 2:**
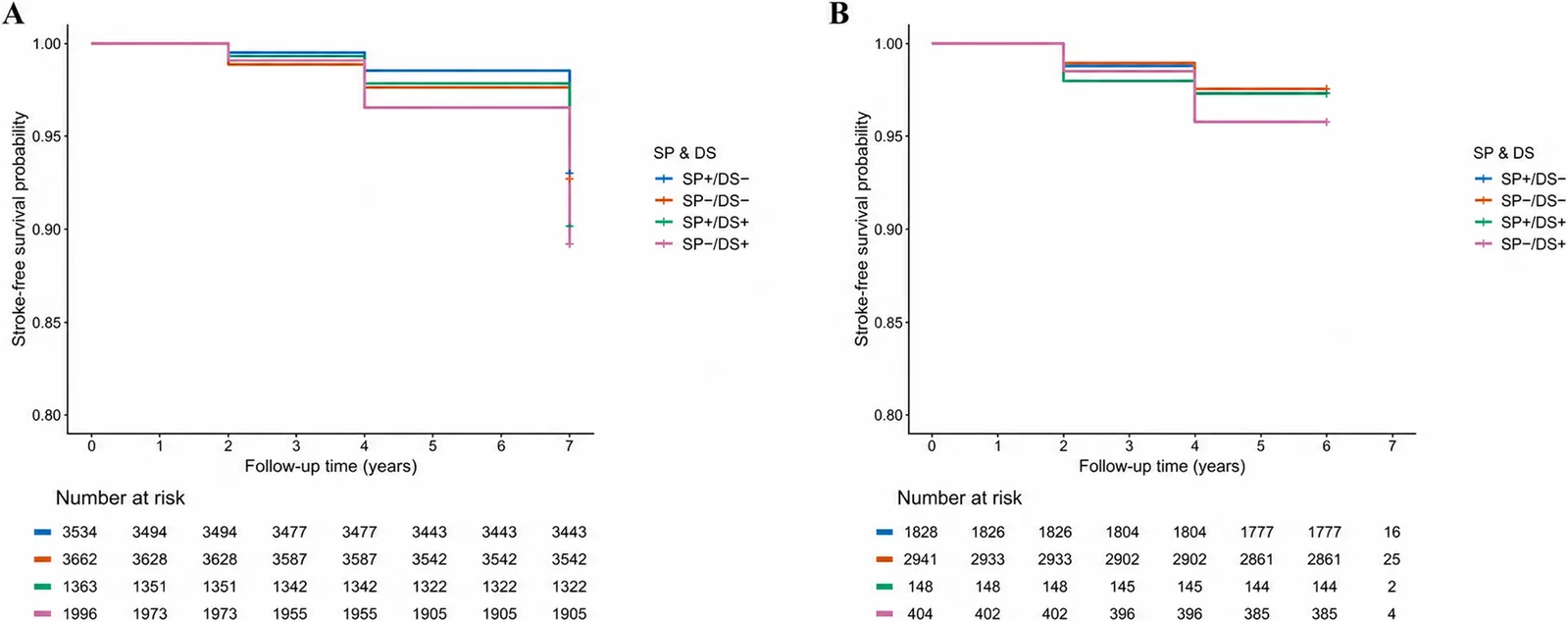
Kaplan–Meier stroke-free survival by SP/DS in CHARLS (**A**) and ELSA (**B**). Kaplan–Meier estimates of stroke-free survival across four baseline psychosocial phenotypes: SP+/DS−, SP−/DS−, SP+/DS+, and SP−/DS+. SP+ indicates participation in ≥ 1 social activity at baseline, SP− indicates no participation in any activity, DS+ indicates CES-D score above the cut-point (CHARLS: CES-D-10 ≥ 10; ELSA: CES-D-8 ≥ 4), DS− indicates below the cut-point

In CHARLS, SP − was not significantly associated with incident stroke (Model 3: HR = 1.14, 95% CI 0.96–1.34, *p* = 0.13), whereas DS + was significantly associated with a higher stroke risk (Model 3: HR = 1.40, 95% CI 1.18–1.65, *p* < 0.001) (Table 2). In ELSA, SP − was likewise not significantly associated with incident stroke (Model 3: HR = 1.05, 95% CI 0.74–1.51, *p* = 0.78). In ELSA, DS + was marginally associated with incident stroke in Model 3 (HR = 1.54, 95% CI 0.95–2.51, *p* = 0.08) (Table 2). Kaplan–Meier curves showed separation in stroke-free survival across the four SP/DS categories in both cohorts (Fig. 2).

**Table 2:** Stroke hazard ratios for social participation and depressive symptoms

Comparison	Adjustment	CHARLS		ELSA	
		HR (95% CI)	*p*	HR (95% CI)	*p*
SP−	Model1	1.11 (0.97, 1.27)	0.14	1.00 (0.74, 1.34)	0.97
	Model2	1.09 (0.94, 1.26)	0.26	1.03 (0.74, 1.43)	0.87
	Model3	1.14 (0.96, 1.34)	0.13	1.05 (0.74, 1.51)	0.78
DS+	Model1	1.48 (1.29, 1.70)	< 0.001	1.36 (0.91, 2.05)	0.14
	Model2	1.49 (1.28, 1.73)	< 0.001	1.53 (0.97, 2.41)	0.07
	Model3	1.40 (1.18, 1.65)	< 0.001	1.54 (0.95, 2.51)	0.08
(SP−/DS−) /(SP+/DS−)	Model1	1.05 (0.89, 1.25)	0.56	0.93 (0.68, 1.28)	0.67
	Model2	1.02 (0.84, 1.23)	0.85	0.92 (0.65, 1.30)	0.63
	Model3	1.03 (0.85, 1.26)	0.74	0.93 (0.64, 1.36)	0.72
(SP+/DS+) /(SP+/DS−)	Model1	1.43 (1.16, 1.77)	< 0.001	1.01 (0.44, 2.33)	0.99
	Model2	1.43 (1.13, 1.80)	0.003	0.74 (0.26, 2.06)	0.56
	Model3	1.40 (1.10, 1.78)	0.006	0.72 (0.25, 2.10)	0.55
(SP−/DS+) /(SP+/DS−)	Model1	1.59 (1.32, 1.90)	< 0.001	1.43 (0.87, 2.33)	0.16
	Model2	1.57 (1.28, 1.93)	< 0.001	1.81 (1.06, 3.11)	0.03
	Model3	1.56 (1.27, 1.93)	< 0.001	1.87 (1.04, 3.37)	0.04

*CHARLS* China Health and Retirement Longitudinal Study, *ELSA* English Longitudinal Study of Ageing, *SP+* participation in ≥ 1 social activity at baseline, *SP −* no participation in any activity, *DS + CES-D* score above the cut-point (CHARLS: CES-D-10 ≥ 10; ELSA: CES-D-8 ≥ 4), *DS−* below the cut-point. *Model 1* unadjusted (crude) model, *Model 2* adjusted for age, sex, marital status, education level, and annual household expenditure, *Model 3* adjusted for Model 2 plus smoking status, alcohol drinking status, BMI, and number of chronic conditions

### Joint associations of SP and DS

In CHARLS, relative to the reference group (SP+/DS−), HRs increased across the combined SP/DS categories: SP−/DS − was not associated with higher stroke risk (Model 3: HR = 1.03, 95% CI 0.85–1.26, *p* = 0.74), SP+/DS + was associated with higher risk (HR = 1.40, 95% CI 1.10–1.77, *p* = 0.006), and SP−/DS + had the highest risk (HR = 1.56, 95% CI 1.27–1.93, *p* < 0.001) (Table 2). In ELSA, neither single-exposure category was significantly associated with stroke risk (SP−/DS−: HR = 0.93, *p* = 0.72; SP+/DS+: HR = 0.72, *p* = 0.55), whereas risk was higher in the joint-exposure group SP−/DS+ (HR = 1.87, 95% CI 1.04–3.37, *p* = 0.04) (Table 2).

### Subgroup and sensitivity analyses

In pre-specified subgroup analyses in CHARLS, the SP−/DS+ group had the highest or near-highest hazard across strata (HR = 1.43–1.85), with larger estimates overall than SP+/DS + and SP−/DS−. Across subgroups, SP−/DS − was not associated with a significant increase in risk, whereas SP+/DS+ reached statistical significance in some strata but with smaller effect sizes than SP−/DS+. The SP−/DS+ association was larger among smokers (HR = 1.85) and participants with BMI ≥ 25 (HR = 1.73), while estimates were attenuated and mostly non-significant among those aged ≥ 65 years; however, formal tests provided no evidence of effect modification. Overall, interaction tests were not statistically significant across stratifying variables (*P* for interaction for SP−/DS+: 0.17–0.63), providing no evidence of statistically significant effect modification, although the direction of the SP−/DS+ estimates was generally consistent across strata (Table 3). In ELSA, subgroup estimates varied across health- and behavior-related strata. Point estimates for SP−/DS + in ELSA generally exceeded 1 across strata, although precision was limited in several subgroups. The estimate was higher among participants with chronic diseases (HR = 2.28, with a wider confidence interval) and reached statistical significance among those with BMI ≥ 25 (HR = 1.94, *p* = 0.04). Conversely, among participants with BMI < 25, SP−/DS − was associated with a lower risk estimate (HR = 0.44, *p* = 0.02), which warrants cautious interpretation. In sex-stratified analyses, SP−/DS + was marginally significant in women (HR = 1.93, *p* = 0.09) and not significant in men. Despite variation in point estimates, interaction tests were not statistically significant (*P* for interaction for SP−/DS+: 0.23–0.84), providing no evidence of statistically significant effect modification (Table 3). However, the direction of the association for the SP−/DS+ group was consistently unfavorable across all strata, indicating a robust risk pattern despite the lack of formal interaction.

**Table 3:** Stratified associations of joint SP/DS phenotype with incident stroke

Comparison	Stratification	Subgroup	CHARLS			ELSA		
			HR (95% CI)	*p*	Interaction_p	HR (95% CI)	*p*	Interaction_p
(SP−/DS−) /(SP+/DS−)	Age	< 65	1.07 (0.86, 1.32)	0.56	0.63	0.62 (0.30, 1.26)	0.19	0.77
		≥ 65	0.97 (0.72, 1.31)	0.86		0.99 (0.66, 1.48)	0.96	
	Chronic	Chronic = 0	1.14 (0.93, 1.41)	0.2	0.15	0.85 (0.56, 1.27)	0.42	0.94
		Chronic ≥ 1	0.86 (0.62, 1.19)	0.36		0.82 (0.42, 1.60)	0.56	
	Drinking	Non-drinker	1.16 (0.93, 1.44)	0.19	0.2	1.80 (0.72, 4.51)	0.21	0.08
		Drinker	0.90 (0.66, 1.24)	0.53		0.66 (0.44, 0.98)	0.04	
	Obesity	BMI < 25	1.05 (0.82, 1.34)	0.71	0.56	0.44 (0.22, 0.87)	0.02	0.39
		BMI ≥ 25	1.17 (0.88, 1.56)	0.28		0.98 (0.62, 1.54)	0.93	
	Sex	Female	1.02 (0.79, 1.33)	0.86	0.79	1.16 (0.67, 2.01)	0.6	0.68
		Male	1.08 (0.85, 1.36)	0.54		0.70 (0.45, 1.11)	0.13	
	Smoking	Non-smoker	1.02 (0.80, 1.29)	0.88	0.64	1.11 (0.61, 2.01)	0.74	0.47
		Smoker	1.11 (0.86, 1.43)	0.44		0.74 (0.48, 1.13)	0.16	
(SP+/DS+) /(SP+/DS−)	Age	< 65	1.55 (1.20, 2.01)	0.001	0.14	0.47 (0.06, 3.64)	0.47	0.97
		≥ 65	1.11 (0.76, 1.60)	0.6		0.88 (0.27, 2.89)	0.84	
	Chronic	Chronic = 0	1.53 (1.19, 1.97)	0.001	0.33	0.75 (0.27, 2.10)	0.59	0.89
		Chronic ≥ 1	1.22 (0.82, 1.80)	0.33		0.00 (0.00, Inf)	1	
	Drinking	Non-drinker	1.46 (1.12, 1.89)	0.004	0.61	0.72 (0.09, 5.74)	0.75	0.72
		Drinker	1.28 (0.84, 1.94)	0.25		0.70 (0.22, 2.29)	0.56	
	Obesity	BMI < 25	1.42 (1.06, 1.91)	0.02	0.82	0.00 (0.00, Inf)	1	0.99
		BMI ≥ 25	1.35 (0.93, 1.96)	0.11		1.12 (0.39, 3.18)	0.84	
	Sex	Female	1.63 (1.23, 2.15)	0.001	0.2	0.34 (0.05, 2.55)	0.29	0.72
		Male	1.21 (0.86, 1.72)	0.28		1.30 (0.40, 4.23)	0.66	
	Smoking	Non-smoker	1.58 (1.21, 2.07)	0.001	0.28	0.00 (0.00, Inf)	1	0.51
		Smoker	1.24 (0.87, 1.76)	0.24		0.87 (0.31, 2.45)	0.79	
(SP−/DS+) /(SP+/DS−)	Age	< 65	1.69 (1.35, 2.11)	0.001	0.17	1.53 (0.66, 3.55)	0.32	0.23
		≥ 65	1.28 (0.93, 1.77)	0.13		1.36 (0.65, 2.86)	0.41	
	Chronic	Chronic = 0	1.66 (1.33, 2.07)	0.001	0.47	1.18 (0.65, 2.15)	0.6	0.73
		Chronic ≥ 1	1.43 (1.03, 1.99)	0.03		2.28 (0.72, 7.20)	0.16	
	Drinking	Non-drinker	1.64 (1.31, 2.06)	0.001	0.52	2.13 (0.70, 6.54)	0.19	0.43
		Drinker	1.43 (0.99, 2.05)	0.05		1.12 (0.58, 2.14)	0.74	
	Obesity	BMI < 25	1.50 (1.15, 1.94)	0.002	0.49	0.52 (0.15, 1.87)	0.32	0.43
		BMI ≥ 25	1.73 (1.25, 2.38)	0.001		1.94 (1.03, 3.66)	0.04	
	Sex	Female	1.57 (1.21, 2.03)	0.001	0.63	1.93 (0.91, 4.10)	0.09	0.84
		Male	1.71 (1.31, 2.24)	0.001		1.13 (0.51, 2.47)	0.77	
	Smoking	Non-smoker	1.48 (1.16, 1.89)	0.002	0.23	0.86 (0.25, 2.97)	0.82	0.31
		Smoker	1.85 (1.40, 2.44)	0.001		1.47 (0.81, 2.68)	0.21	

*CHARLS* China Health and Retirement Longitudinal Study, *ELSA* English Longitudinal Study of Ageing, *BMI* Body Mass Index, *SP+* participation in ≥ 1 social activity at baseline, *SP −* no participation in any activity, *DS + CES-D* score above the cut-point (CHARLS: CES-D-10 ≥ 10; ELSA: CES-D-8 ≥ 4), *DS−* below the cut-point

Results from the multiple-imputation sensitivity analysis were broadly consistent with the primary analysis in ELSA: after imputation, DS + was significantly associated with incident stroke (Model 3: HR = 1.57, 95% CI 1.02–2.43, *p* = 0.04), and the SP−/DS+ group remained at significantly higher risk (HR = 1.85, 95% CI 1.09–3.13, *p* = 0.02); findings in CHARLS were also unchanged (SP−: HR = 1.10, *p* = 0.15; DS+: HR = 1.40, *p* < 0.001; SP−/DS+: HR = 1.49, *p* < 0.001) (Table 4).

**Table 4:** Multiple-imputation sensitivity of SP/DS phenotypes for stroke risk

Comparison	Adjustment	CHARLS		ELSA	
		HR (95% CI)	*p*	HR (95% CI)	*p*
SP−	Model1	1.09 (0.96, 1.24)	0.19	1.01 (0.76, 1.35)	0.93
	Model2	1.08 (0.95, 1.23)	0.23	1.04 (0.76, 1.43)	0.79
	Model3	1.10 (0.97, 1.26)	0.15	1.03 (0.75, 1.42)	0.86
DS+	Model1	1.42 (1.25, 1.62)	< 0.001	1.43 (0.97, 2.13)	0.07
	Model2	1.41 (1.23, 1.60)	< 0.001	1.63 (1.06, 2.50)	0.03
	Model3	1.40 (1.23, 1.60)	< 0.001	1.57 (1.02, 2.43)	0.04
(SP−/DS−) /(SP+/DS−)	Model1	1.02 (0.87, 1.20)	0.8	0.94 (0.69, 1.28)	0.7
	Model2	1.01 (0.86, 1.19)	0.87	0.92 (0.66, 1.29)	0.64
	Model3	1.04 (0.88, 1.23)	0.67	0.91 (0.64, 1.28)	0.58
(SP+/DS+) /(SP+/DS−)	Model1	1.34 (1.10, 1.64)	0.004	1.00 (0.43, 2.30)	0.99
	Model2	1.31 (1.07, 1.61)	0.009	0.72 (0.26, 2.02)	0.54
	Model3	1.29 (1.05, 1.60)	0.02	0.71 (0.25, 2.01)	0.52
(SP−/DS+) /(SP+/DS−)	Model1	1.51 (1.27, 1.79)	< 0.001	1.54 (0.96, 2.47)	0.08
	Model2	1.49 (1.25, 1.77)	< 0.001	1.95 (1.17, 3.25)	0.01
	Model3	1.49 (1.24, 1.79)	< 0.001	1.85 (1.09, 3.13)	0.02

*CHARLS* China Health and Retirement Longitudinal Study, *ELSA* English Longitudinal Study of Ageing, *SP+* participation in ≥ 1 social activity at baseline, *SP −* no participation in any activity, *DS + CES-D* score above the cut-point (CHARLS: CES-D-10 ≥ 10; ELSA: CES-D-8 ≥ 4), *DS−* below the cut-point, *Model 1* unadjusted (crude) model, *Model 2* adjusted for age, sex, marital status, education level, and annual household expenditure, *Model 3* adjusted for Model 2 plus smoking status, alcohol drinking status, BMI, and number of chronic conditions

## Discussion

Using two nationally representative longitudinal ageing cohorts—the China Health and Retirement Longitudinal Study (CHARLS) and the English Longitudinal Study of Ageing (ELSA)—we assessed the independent and joint associations of social participation (SP) and depressive symptoms (DS) with incident stroke [37]. Across both cohorts, stroke risk was most consistently elevated in the psychosocial phenotype characterised by insufficient social participation with coexisting depressive symptoms (SP−/DS+) [24]. This cross-cohort risk stratification suggests that, for primary stroke prevention, jointly assessing depressive symptoms and social participation may capture risk heterogeneity more effectively than evaluating the average main effect of social participation alone [5, 6, 38].

The observed association between depressive symptoms and incident stroke risk is consistent with robust prospective evidence from existing literature. Meta-analyses of cohort studies have documented that depression is associated with elevated risks of incident stroke and stroke-related mortality [12, 13], and subsequent syntheses that restrict to populations free of baseline cardiovascular and cerebrovascular disease have reached similar conclusions [39]. In our analyses, the association between DS + and stroke risk was numerically stronger in CHARLS, whereas in ELSA, it became more evident after multiple imputation. Importantly, the imputed results serve as supportive sensitivity evidence, confirming that the findings for DS-related phenotypes and the SP−/DS+ subgroup remained robust after accounting for missing data. Consistent with established recommendations [40–42], complete-case Cox models were retained as the primary analyses.

Regarding social factors, previous studies have predominantly focused on indicators of “relationship-deficit”, such as social isolation and loneliness, which are increasingly recognized as determinants of cardiovascular and brain health [25]. Prospective evidence indicates that deficiencies in social relationships are associated with elevated risks of coronary heart disease and stroke [32]. However, social exposures are heterogeneous: loneliness, isolation, and participation behaviors capture overlapping yet distinct constructs. Their associations with health outcomes may attenuate after adjustment for comorbidity and health behaviors, partly because social participation is closely intertwined with baseline health status and broader psychosocial risk [32, 43]. Against this backdrop, our behaviour-oriented definition of insufficient social participation did not show a consistent main association with stroke across cohorts. Nevertheless, a clearer high-risk profile emerged when DS co-occurred within the SP−/DS+ phenotype. This pattern underscores the importance of jointly assessing social participation together and psychological distress in a combined phenotype framework, rather than interpreting social participation as an isolated average exposure effect [25, 32].

One possible explanation for the observed risk concentration is that depressive symptoms and insufficient social participation capture overlapping yet distinct vulnerabilities. Specifically, depressive symptoms may contribute to vascular risk through several interrelated biological pathways, including inflammation, neuroendocrine dysregulation, and endothelial dysfunction [22, 44–48]. Concurrently, social participation may provide emotional and instrumental support, stress-buffering resources, and reinforcement of health-related routines [43, 49]. Among individuals with insufficient social participation, such protective resources are diminished, potentially reducing engagement in preventive behaviors, timely help-seeking, and sustained risk-factor control [4, 50]. In that sense, the SP−/DS+ phenotype may identify individuals with coexisting psychological vulnerability and limited social resources. This renders integrated prevention strategies, combining mental health assessment with social-connection and behavior-support, particularly relevant for this high-risk group [4, 25].

Even in the absence of a statistically significant multiplicative interaction, the four-category phenotype retains risk-stratification utility by helping to identify subgroups with concentrated risk [4, 50]. We observed distinct joint-exposure patterns across the two cohorts: CHARLS exhibited a more graded increase in stroke risk across categories, whereas in ELSA, the risk elevation was primarily concentrated in the SP−/DS+ subgroup. These cross-cohort discrepancies may reflect variations in how social participation is embedded in community life, primary care, and social-service systems, which can influence whether social activity participation translates into sustained support and resource linkage [25, 43]. In addition, the assessment of depressive symptoms relied on different CES-D versions and cohort-specific cut-offs (CHARLS: CES-D-10; ELSA: CES-D-8), a methodological difference that should be considered when interpreting variations in DS+ prevalence and effect estimates [51, 52]. Nevertheless, the SP−/DS+ phenotype consistently demonstrated a higher-risk pattern in both cohorts.

These findings may inform primary stroke prevention. Contemporary stroke prevention strategies increasingly advocate for comprehensive life-course risk management [4], and cardiovascular health frameworks likewise emphasize holistic, integrated risk control [50]. Our results suggest that structured psychosocial assessment may help identify older adults with coexisting depressive symptoms and insufficient social participation, a profile that may warrant closer preventive attention [44, 47, 49, 53]. This joint profile could inform bundled strategies that pair depression screening and management with efforts to strengthen social connection and reinforce healthy routines, including community referral models and social prescribing where appropriate [25, 54].

Several limitations should be noted. First, stroke cases were ascertained through self-reported physician diagnosis, which may introduce outcome misclassification. Although validation evidence supports the use of self-reported stroke in epidemiological research, non-differential misclassification could attenuate associations. Furthermore, differential reporting bias related to depressive symptoms, healthcare use, or recall cannot be fully excluded [55, 56]. Second, social participation was measured using a harmonized binary indicator available in both cohorts, distinguishing participation in at least one social, leisure, or group activity from no participation; however, this measure did not capture participation frequency, intensity, activity type, or potential dose–response patterns. Further activity-count or activity-type analyses would require cohort-specific reconstruction from item-level social activity variables, and because the activity items and their sociocultural meanings differ between CHARLS and ELSA, such analyses could introduce additional measurement non-comparability. Thirdly, depressive symptoms were assessed using different CES-D versions and cohort-specific cut-offs, which may limit the direct comparability of prevalence estimates across the two cohorts [51]. Despite these measurement differences, the SP−/DS+ phenotype showed a higher-risk pattern in both cohorts, supporting the robustness of the main finding. Residual confounding, reverse causation from subclinical cerebrovascular disease, wave-level approximation of event timing, and limited event numbers in some ELSA subgroups may also have affected the precision and interpretation of the estimates. Finally, the behavioral, service-access, and biological pathways proposed herein remain hypothetical. Future studies with repeated measurements of psychosocial status and health behaviors are warranted to validate these mechanistic pathways and further unpack how the SP−/DS+ phenotype contributes to incident stroke.

In summary, across two nationally representative ageing cohorts, the psychosocial phenotype characterized by insufficient social participation co-occurring with depressive symptoms (SP−/DS+) was associated with higher incident stroke risk, although the precision and pattern of estimates differed between cohorts. These findings support considering joint psychosocial assessment in stroke-prevention risk stratification, while further studies are needed to clarify mechanisms and evaluate prevention strategies.

## Conclusions

Across two nationally representative ageing cohorts (CHARLS 2011–2018 and ELSA 2012–2019), depressive symptoms (DS) were associated with a higher risk of incident stroke, with the association most evident in CHARLS and directionally consistent in ELSA. In contrast, social participation (SP) considered alone showed limited differences in stroke risk after accounting for key covariates. However, when SP was evaluated jointly with DS, a clear and reproducible high-risk phenotype emerged: participants with both insufficient SP and DS (SP−/DS+) consistently had the highest stroke risk compared with the reference group (SP+/DS−) (CHARLS: HR 1.56, 95% CI 1.27–1.93; ELSA: HR 1.87, 95% CI 1.04–3.37, in fully adjusted models). These findings highlight the value of integrating depression assessment with social-functioning evaluation to improve psychosocial risk stratification and inform targeted, prevention-oriented strategies for stroke among older adults.

## Data Availability

This study used publicly available, de-identified data. The original data collection for ELSA and CHARLS received ethical approval from their respective institutional review boards. More information regarding obtaining data for research use can be found at the CHARLS database (https://charls.charlsdata.com/pages/data/111/en.html) and ELSA database (https://www.elsa-project.ac.uk/accessing-elsa-data).

## Abbreviations

DS: Depressive symptoms
SP: Social participation
CHARLS: China Health and Retirement Longitudinal Study
ELSA: English Longitudinal Study of Ageing
CES-D-10: 10-item Center for Epidemiologic Studies Depression Scale
CES-D-8: 8-item Center for Epidemiologic Studies Depression Scale
HR: Hazard ratio
BMI: Body mass index
CI: Confidence intervals
